# Supplementary material for: Barents-Kara sea-ice decline attributed to surface warming in the Gulf Stream
Source: Nat Commun. 2022 Jul 15;13:3767. doi: 10.1038/s41467-022-31117-6 (PMC9287400; doi:10.1038/s41467-022-31117-6)
Supplement: Supplementary file 1 — Supplementary Information [file 41467_2022_31117_MOESM1_ESM.pdf]

Supplementary information

Barents-Kara sea-ice decline attributed to surface warming in the Gulf Stream

Yoko Yamagami[1*], Masahiro Watanabe[2], Masato Mori[3], & Jun Ono[1]

1: Japan Agency for Marine-Earth Science and Technology, Yokohama, Japan

2: Atmosphere and Ocean Research Institute, University of Tokyo, Kashiwa, Japan

3: Research Institute for Applied Mechanics, Kyushu University, Kasuga, Japan

* Corresponding author:
Yoko Yamagami, Research Center for Environmental Modeling and Application, Japan Agency for Marine-Earth Science and Technology, 3173-25 Showamachi, Kanazawaku, Yokohama, Kanagawa 236-0001, Japan
E-mail: y.yamagami@jamstec.go.jp

Contents of this supplementary information

Supplementary Figures 1 to 10.

Supplementary Tables 1 to 2.

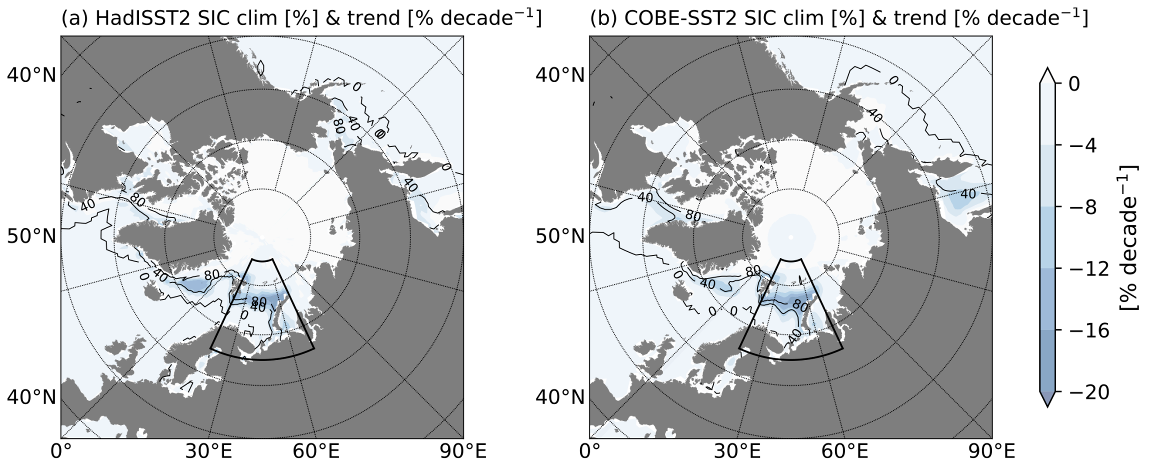

Supplementary Figure 1. | Observed winter climatological SIC and linear trend in the Arctic Ocean for 1970–2017. Climatological DJF mean SIC [%] (black contours) and linear trends of DJF mean SIC [% decade^{-1}] (blue colors) for **(a)** HadISST2 and **(b)** COBE-SST2. The contour intervals of SIC is 40 [%]. The area enclosed by black solid lines is defined as the Barents-Kara Sea in this study.

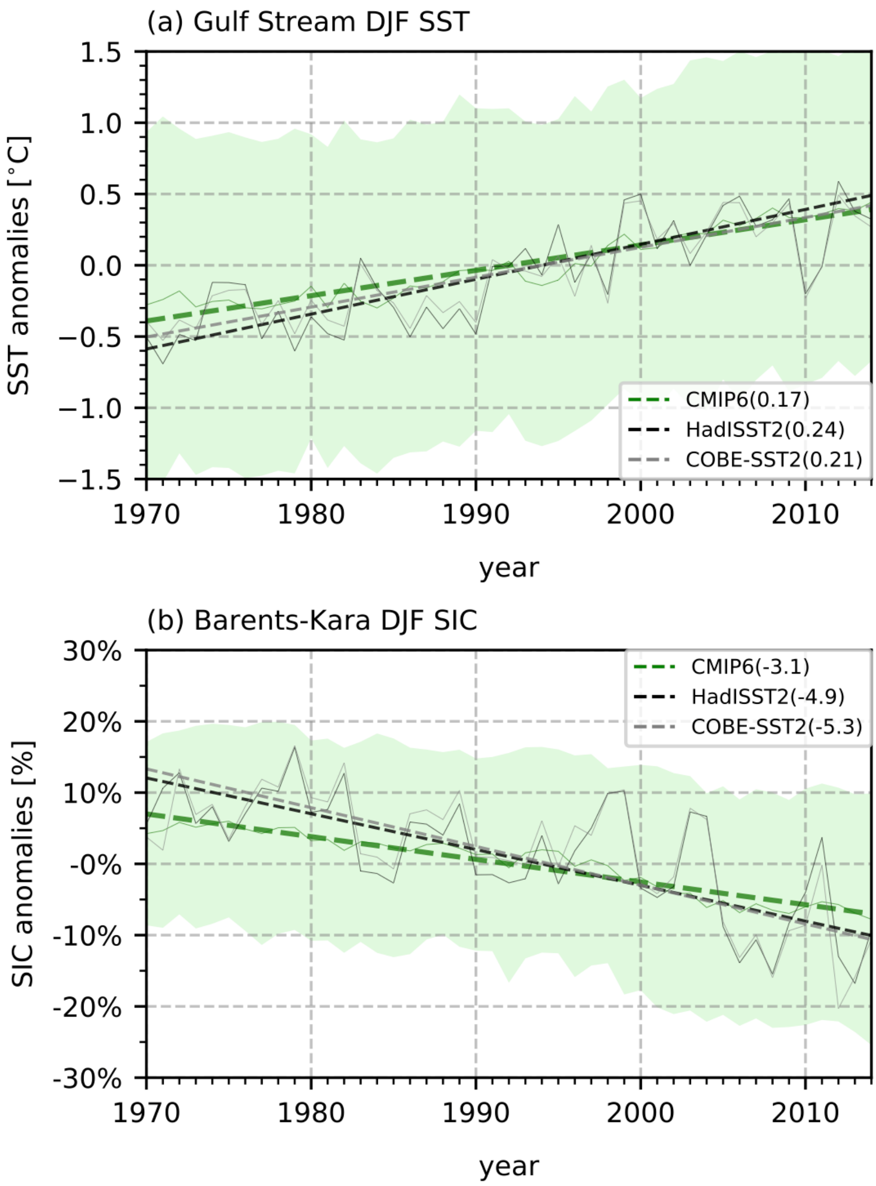

Supplementary Figure 2. | Observed and simulated time series of winter SST in the Gulf Stream regions and SIC in the Barents-Kara Sea. a and **b.** As in **Figure 1a** and **b**, but for single members of the 39 CMIP6 models (Supplementary Table 1).

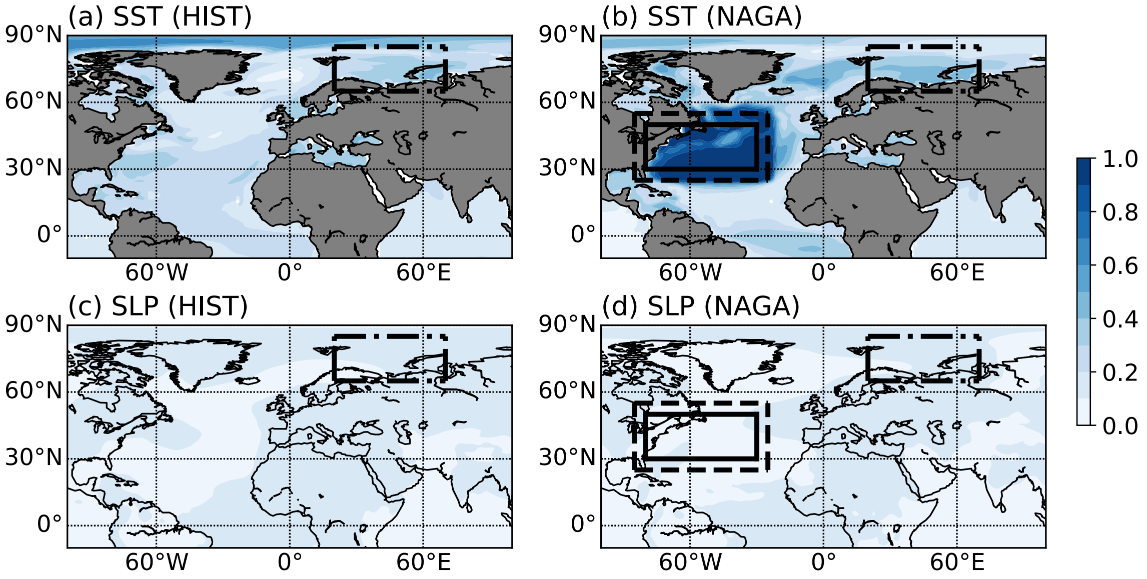

Supplementary Figure 3. | Ratio of ensemble mean variance to the total ensemble member variance for SST and SLP in the Atlantic sector of the Northern Hemisphere. Variance ratio (Eq. 2; Methods) for SST over the North Atlantic in **(a)** HIST and **(b)** NAGA. The dash-dot box indicates the Barents-Kara Sea. The solid box shows the area in which SST anomalies are fully restored to the observed values. The restored SST anomalies are linearly reduced to 0 from the solid box to the dashed box. **c** and **d**. As in **a-b,** but for SLP.

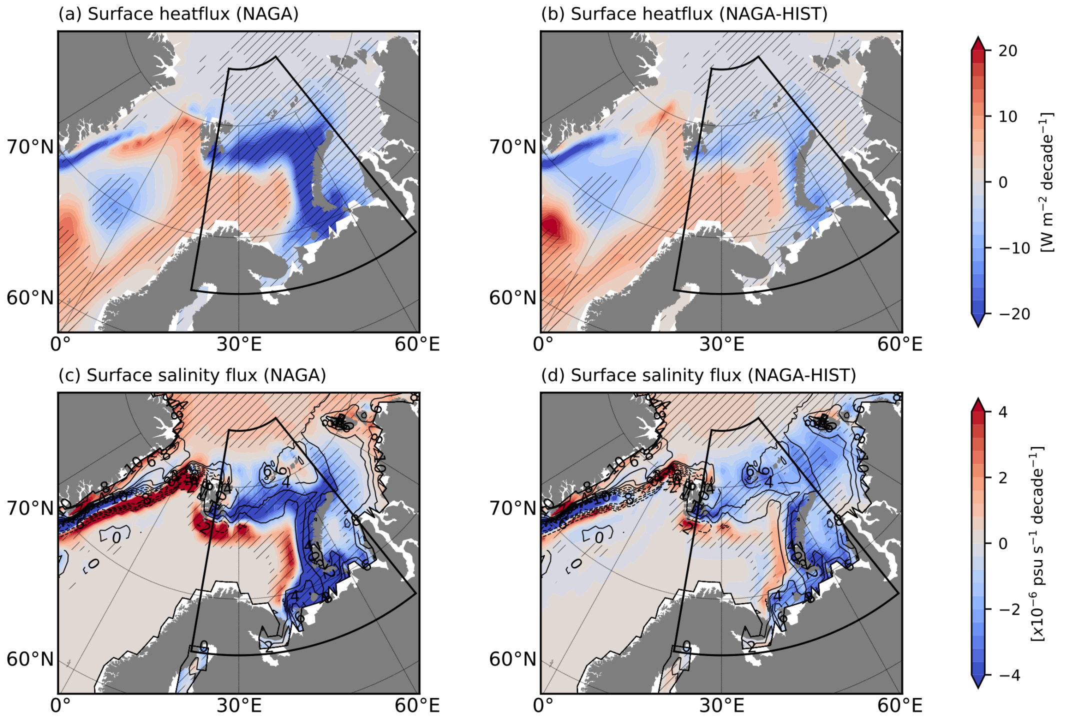

Supplementary Figure 4. | Linear trends of surface fluxes over the Barents-Kara Sea. As in Fig. 2, but for **(a)** surface heat flux from the atmosphere to the ocean [Wm^{-2} $decade^{-1}$] and **(b)** the difference in the Barents-Kara Sea. **c,d** As in **a,b** but for salinity flux [$\times 10^{-6}$psu s^{-1} $decade^{-1}$]. The climatology of salinity flux [$\times 10^{-4}$ psu s^{-1}] in NAGA is shown by contours in **(c)** and **(d)**. The contour interval is 2.

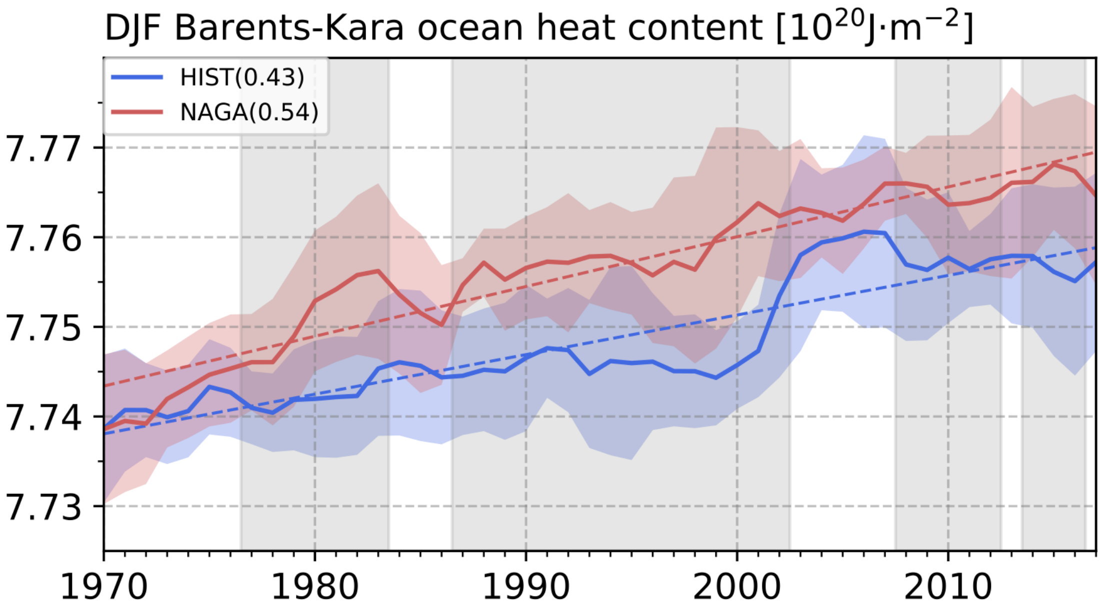

Supplementary Figure 5. | Simulated time series of the heat content averaged over the Barents-Kara Sea. DJF mean surface heat content [10^{20} J m^{-2}] (solid line) and linear trends [10^{20} J m^{-2} $decade^{-1}$] (dashed line) averaged over the Barents-Kara Sea for HIST (blue) and NAGA (red). The heat content is vertically integrated from the surface to a depth of 345m. Red and blue shades indicate ±1 standard deviations for 10 ensemble members in both experiments. The numbers of trends are shown in the legend. The gray shaded area indicates a period when the difference between HIST and NAGA was statistically significant based on a two-tailed Student's t-test at the 95% confidence level.

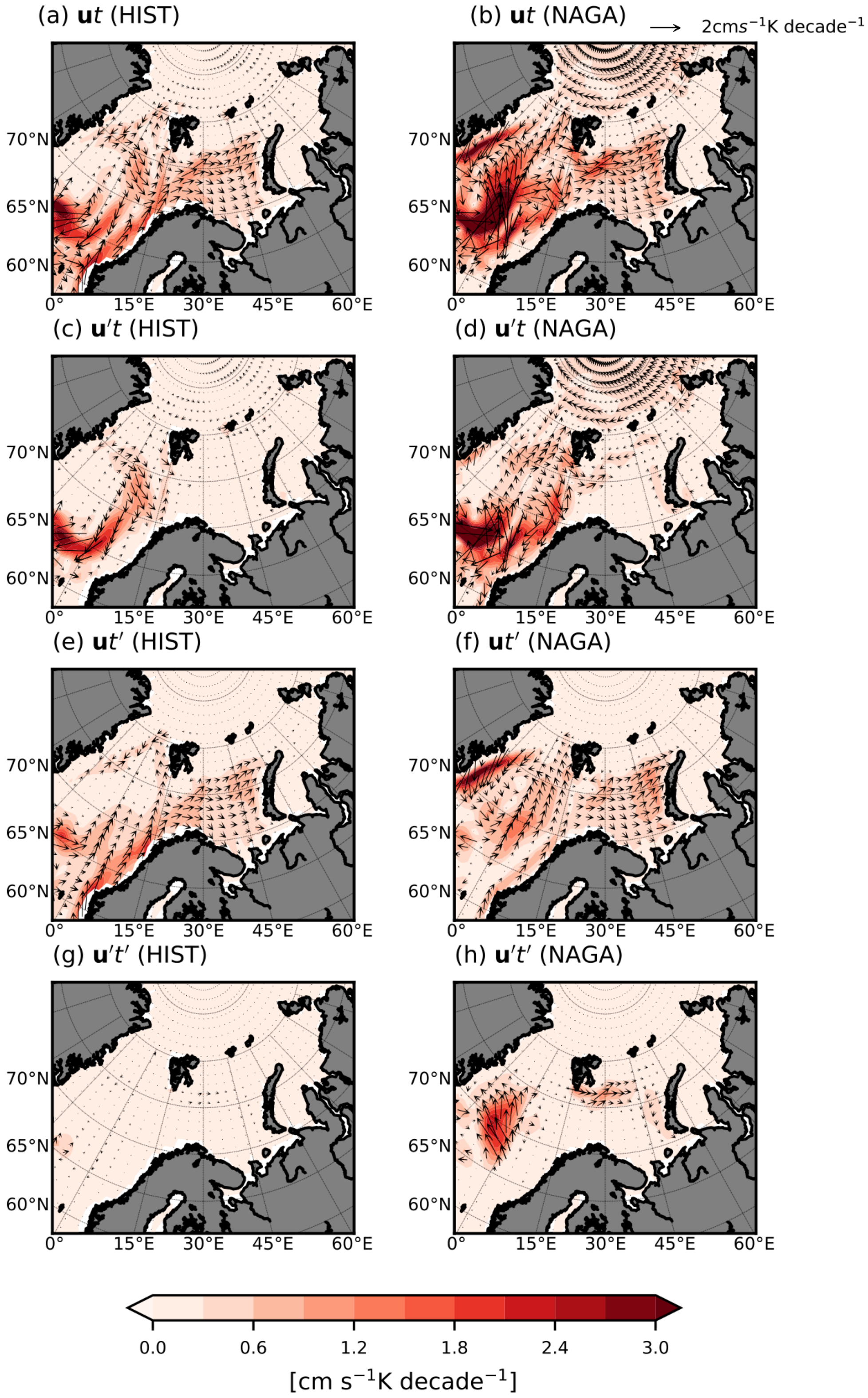

(a) **u***t* (HIST)
(b) **u***t* (NAGA)
2cms^{-1}K decade^{-1}
(c) **u**′*t* (HIST)
(d) **u**′*t* (NAGA)
(e) **u***t*′ (HIST)
(f) **u***t*′ (NAGA)
(g) **u**′*t*′ (HIST)
(h) **u**′*t*′ (NAGA)
70°N
65°N
60°N
0°
15°E
30°E
45°E
60°E
0.0
0.6
1.2
1.8
2.4
3.0
[cm s^{-1}K decade^{-1}]

46

Supplementary Figure 6. | The decomposed contribution of each term to the horizontal heat flux trends defined in Eq. (4). Linear trends of the DJF horizontal heat flux ($\mathbf{u}t$) at 54 m (vector) over 10 years for **(a)** HIST and **(b)** NAGA. The magnitude of the vector is shown by color. **c-h**. As in **a** and **b,** but for the contributions of **(c)** and **(d)** velocity trends ($\mathbf{u}'t$), **(e)** and **(f)** temperature trends ($\mathbf{u}t'$), and **(g) and (h)** covariability between temperature and velocity trends ($\mathbf{u}'t'$).

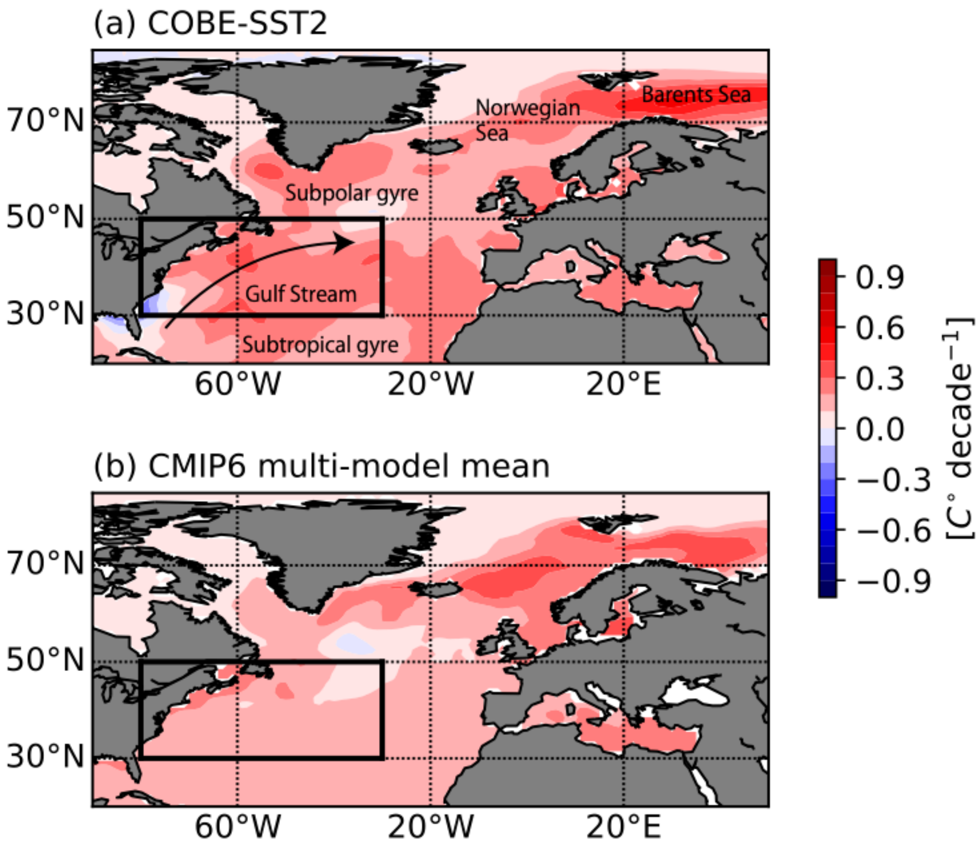

Supplementary Figure 7. | Observed and simulated linear trends of winter SST in the North Atlantic for 1970-2014. Linear trend of DJF mean SST [°C decade^{-1}] in **(a)** COBE-SST2 and **(b)** the multimodel mean of 39 CMIP6 models.

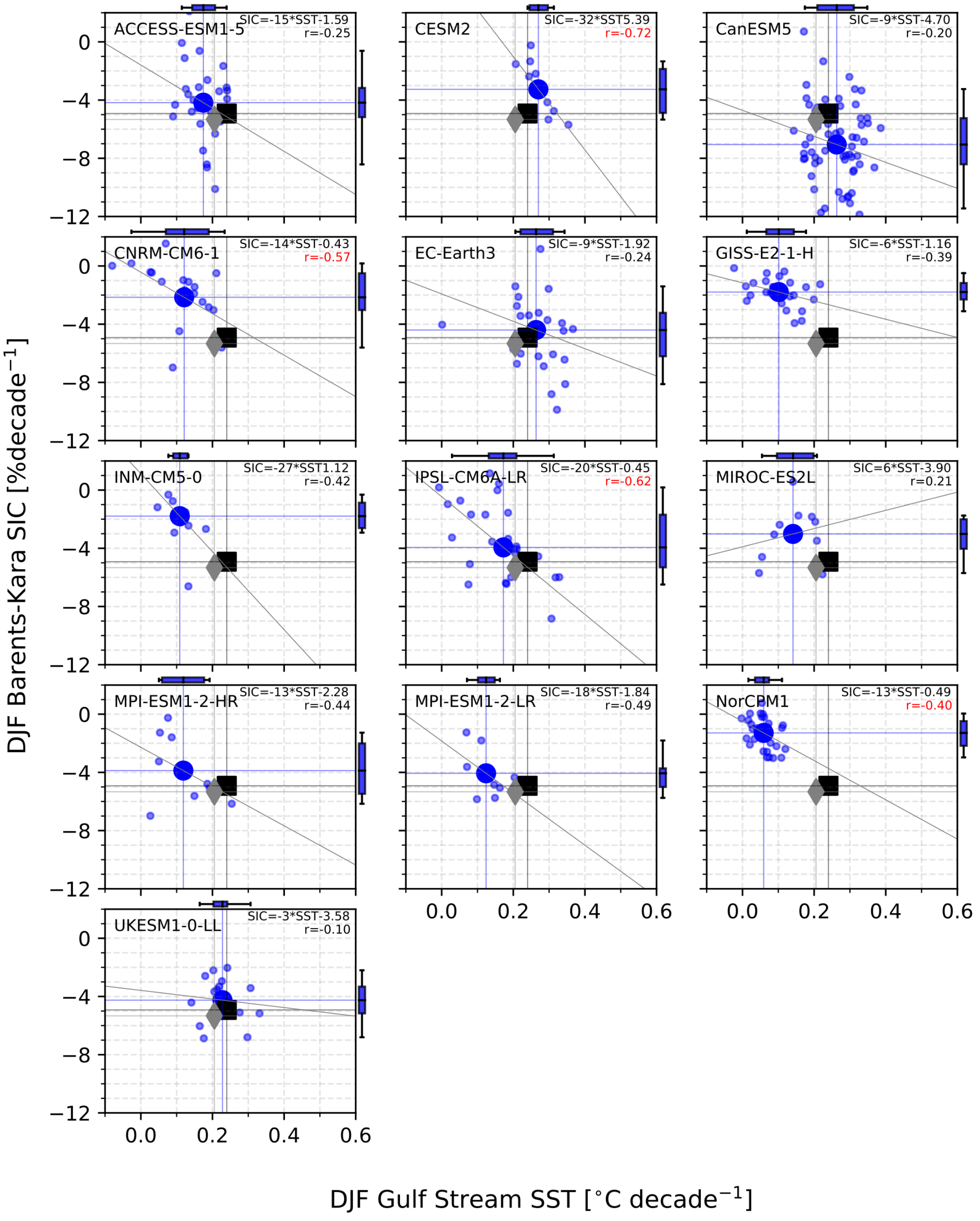

Supplementary Figure 8. | Relationships between the linear trends (1970–2014) of SIC and SST in each CMIP6 model. As in **Figure 6b,** but for CMIP6 models with

more than ten available ensemble members. If the correlation coefficient is statistically significant at the 95% confidence level, the correlation is shown in red.

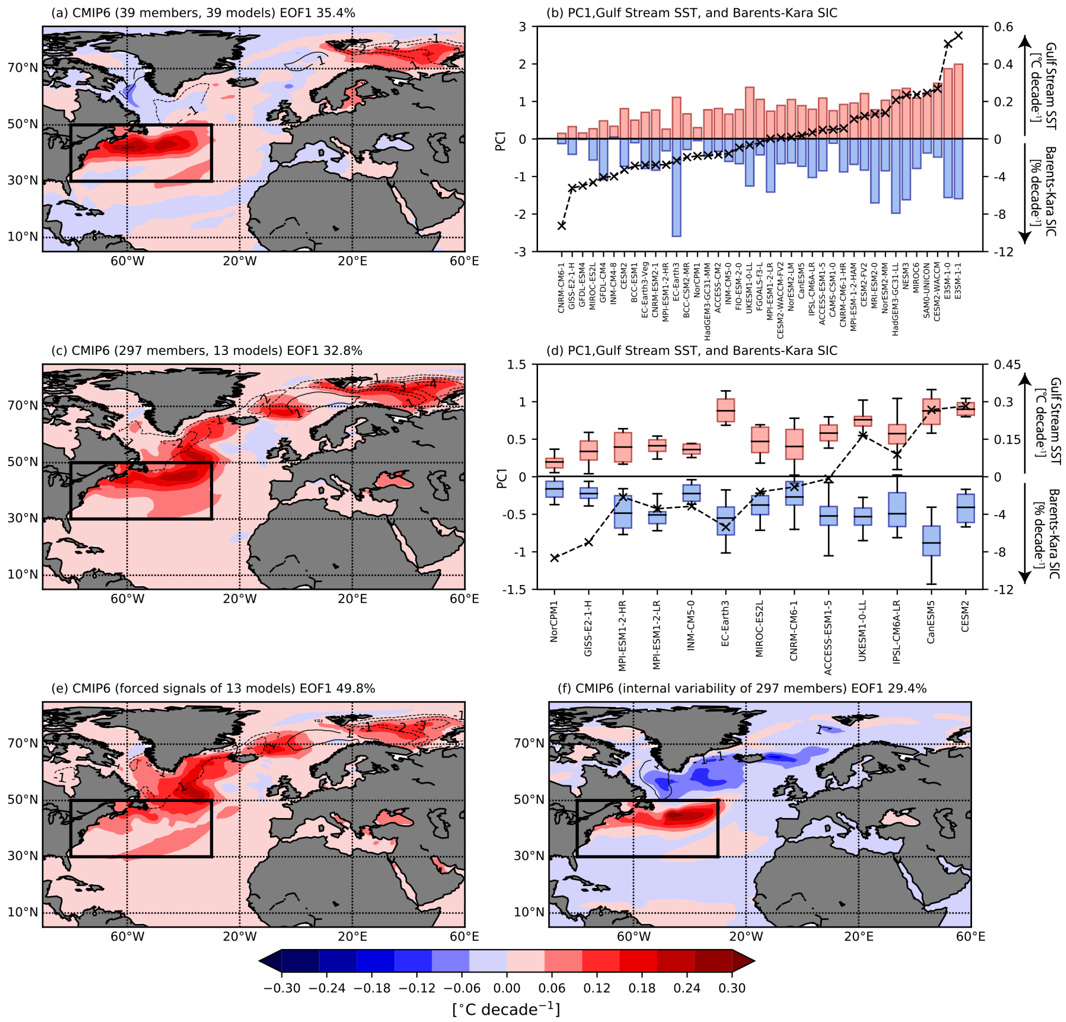

Supplementary Figure 9. | Intermodel variations in SST trends in CMIP6 models and composites of SST trends for CMIP6 climate models and MIROC6 experiments

a. Anomaly of SST (color) and SIC (contour) trends regressed to the principal component of the first EOF mode based on 39 CMIP6 historical simulations from 1970-2014. The explained variance of the first EOF mode is marked at the top right of the panel. The area used for the EOF analysis is shown in the black box. **b.** Principal components of the first EOF mode (line plot). Red (blue) bars indicate the Gulf Stream

SST (Barents-Kara SIC) trend. **c** and **d.** As in **a** and **b,** but for all ensemble members of the CMIP6 model that have more than ten ensemble members available. The averages of the principal components of the first EOF for each model are shown with line plots. The red (blue) box plot indicates the Gulf Stream SST (Barents-Kara SIC) trend for each model. **e** and **f.** As in **c,** but for **(e)** the ensemble means of 13 models (i.e., externally forced SST trends) and **(f)** deviations from the ensemble means of 297 members of 13 models (i.e., internal variability of SST trends).

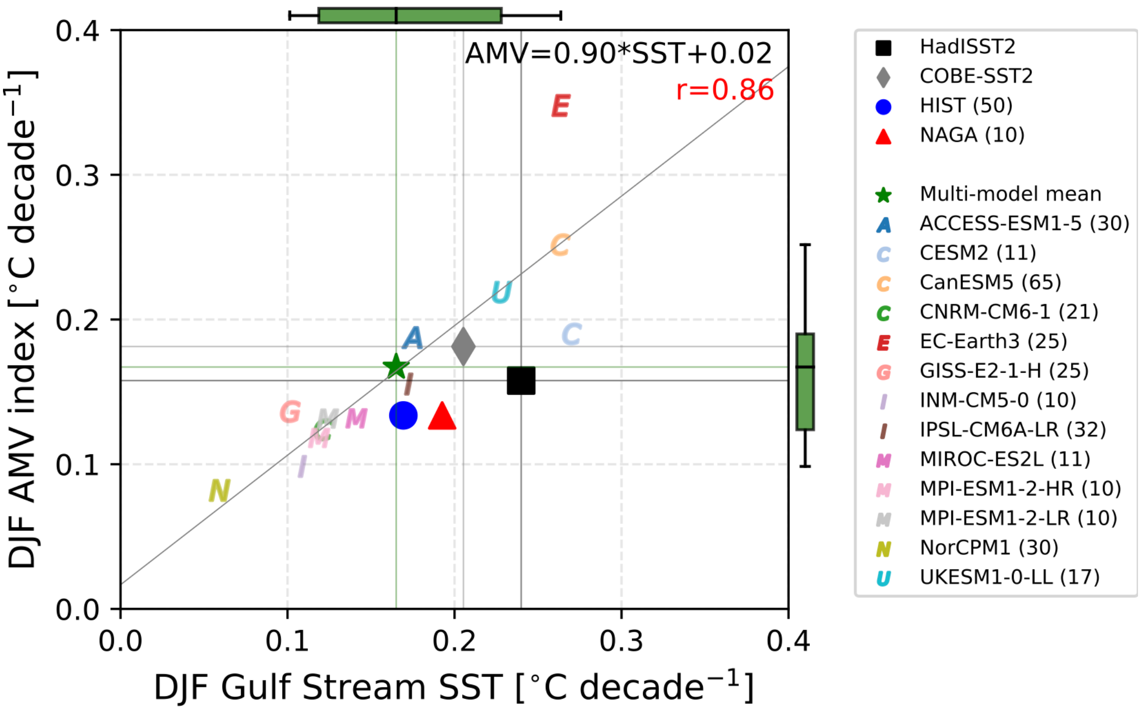

Supplementary Figure 10. | Relationships between the linear trends (1970-2014) of the AMV index and Gulf Stream SST for HIST, NAGA, CMIP6, and observations. As in **Figure 6c,** but for the DJF mean AMV index and Gulf Stream SST.

Supplementary Table 1. | List of 39 CMIP6 historical simulations used in this study. List of model names and variant labels used in this study.

Model Name	Run type
ACCESS-CM2	r1i1p1f1
ACCESS-ESM1-5	r1i1p1f1
BCC-CSM2-MR	r1i1p1f1
BCC-ESM1	r1i1p1f1
CAMS-CSM1-0	r1i1p1f1
CanESM5	r1i1p1f1
CESM2	r1i1p1f1
CESM2-FV2	r1i1p1f1
CESM2-WACCM	r1i1p1f1
CESM2-WACCM-FV2	r1i1p1f1
CNRM-CM6-1	r1i1p1f2
CNRM-CM6-1-HR	r1i1p1f2
CNRM-ESM2-1	r1i1p1f2
E3SM-1-0	r1i1p1f1
E3SM-1-1	r1i1p1f1
EC-Earth3	r1i1p1f1
EC-Earth3-Veg	r1i1p1f1
FGOALS-f3-L	r1i1p1f1
FIO-ESM-2-0	r1i1p1f1
GFDL-CM4	r1i1p1f1
GFDL-ESM4	r1i1p1f1
GISS-E2-1-H	r1i1p1f1
HadGEM3-GC31-LL	r1i1p1f3
HadGEM3-GC31-MM	r1i1p1f3
INM-CM4-8	r1i1p1f1
INM-CM5-0	r1i1p1f1
IPSL-CM6A-LR	r1i1p1f1
MIROC-ES2L	r1i1p1f2
MIROC6	r1i1p1f1
MPI-ESM-1-2-HAM	r1i1p1f1
MPI-ESM1-2-HR	r1i1p1f1
MPI-ESM1-2-LR	r1i1p1f1
MRI-ESM2-0	r1i1p1f1
NESM3	r1i1p1f1
NorCPM1	r1i1p1f1
NorESM2-LM	r1i1p1f1
NorESM2-MM	r1i1p1f1
SAM0-UNICON	r1i1p1f1
UKESM1-0-LL	r1i1p1f2

Supplementary Table 2. | List of CMIP6 models with more than 10 ensemble members. List of model names and the number of ensemble members used in this study.

Model Name	The number of ensemble members
ACCESS-ESM1-5	30
CESM2	11
CanESM5	65
CNRM-CM6-1	21
EC-Earth3	25
GISS-E2-1-H	25
INM-CM5-0	10
IPSL-CM6A-LR	32
MIROC-ES2L	11
MPI-ESM1-2-HR	10
MPI-ESM1-2-LR	10
NorCPM1	30
UKESM1-0-LL	17
